# A Comparison of Sensorimotor Adaptation in the Visual and in the Auditory Modality

**DOI:** 10.1371/journal.pone.0107834

**Published:** 2014-09-25

**Authors:** Gerd Schmitz, Otmar Bock

**Affiliations:** 1 Institute of Physiology and Anatomy, German Sport University, Köln, Germany; 2 Institute of Sports Science, Leibniz University Hannover, Hannover, Germany; Ludwig-Maximilian University, Germany

## Abstract

We compared sensorimotor adaptation in the visual and the auditory modality. Subjects pointed to visual targets while receiving direct spatial information about fingertip position in the visual modality, or they pointed to visual targets while receiving indirect information about fingertip position in the visual modality, or they pointed to auditory targets while receiving indirect information about fingertip position in the auditory modality. Feedback was laterally shifted to induce adaptation, and aftereffects were tested with both target modalities and both hands. We found that aftereffects of adaptation were smaller when tested with the non-adapted hand, i.e., intermanual transfer was incomplete. Furthermore, aftereffects were smaller when tested in the non-adapted target modality, i.e., intermodal transfer was incomplete. Aftereffects were smaller following adaptation with indirect rather than direct feedback, but they were *not* smaller following adaptation with auditory rather than visual targets. From this we conclude that the magnitude of adaptive recalibration rather depends on the method of feedback delivery (indirect versus direct) than on the modality of feedback (visual versus auditory).

## Introduction

Visuo-motor adaptation is typically examined by asking subjects to point with their hand at visual targets while visual feedback of the hand is distorted. Pointing accuracy deteriorates when the distortion is introduced and then gradually recovers. Upon removal of the distortion aftereffects emerge. They are typically measured during movements with undistorted or even without feedback, and are interpreted as recalibration of sensorimotor transformation rules, unbiased by strategic adjustments [1]. Aftereffects transfer between visual and auditory modality [2]–[5], thus it might be that adaptive mechanisms are independent from sensory modalities and receive un-weighted sensory input [6]. But we have recently observed that aftereffects are smaller when they are assessed with auditory rather than with visual targets [7], and proposed two interpretations for this finding: aftereffects either are smaller in the auditory than in the visual modality, or they are smaller in an unpracticed than in the previously practiced modality. A distinction between these two alternatives was not possible in our previous study, because the auditory modality was always the unpracticed one. The present work overcomes this problem by exposing some subjects to a visuo-motor and others to an audio-motor distortion, and then testing for aftereffects in all subjects both with visual and with auditory targets, in counterbalanced order.

Perceptual auditory adaptation might be limited to sensitive periods in development [8]–[9], but adults can adapt to an audio-motor discordance, for example when they have previously learned to accurately shape hand apertures in response to auditory information about object sizes [10]. In contrast to visuo-motor adaptation, audio-motor adaptation has rarely been evaluated in literature. It is indeed challenging to establish a method that delivers distorted auditory feedback of reaching movements; previous authors either used pseudophones (rotatable pair of microphones placed on the subjects’ head at interaural distance and connected to a pair of headphones) or a feedback loudspeaker positioned near the index-fingertip after each pointing response. The former approach produced no aftereffects [5], possibly because sound delivery was cumbersome and thus didn’t encourage lasting adaptation. The latter approach led to robust aftereffects with visual targets [2], but a comparison between visual and auditory aftereffects was not undertaken. Furthermore, the terminal feedback provided in the latter study is difficult to compare with the continuous feedback typically provided in research on visuo-motor adaptation.

Recently Boyer et al. [11] designed a new feedback method as they transformed target and hand positions into auditory avatars (white noise spatialized by Head-Related Transfer Functions). Pointing accuracy was not altered by auditory feedback but by target presentation time, suggesting that the sound was concise enough to display stationary positions, but not positional changes or movements. Oscari et al. [12] provided real-time auditory feedback about Cartesian error during one-dimensional reaching movements. Subjects adapted first to a force, which was suddenly applied perpendicular to the intended movement direction, while they received spatially veridical visual or auditory feedback, and then to a spatial distortion of this feedback. Both feedback methods induced similar aftereffects of the force field; however, force field adaptation relies heavily on proprioceptive feedback [13], and the contributions of auditory versus visual feedback are therefore difficult to compare. Transfer to the unpracticed modality and aftereffects of the kinematic distortions were not tested by Oscari et al. [12].

The present study uses auditory feedback in a slightly different fashion than Oscari et al. [12]. Hand position is coded by a tone that comes from the left or right when the hand is too far to the left or right, respectively; the pitch of this tone decreases as the hand approaches the required movement direction, and an explosion sounds when the hand reaches that position. This together approximates the noise of an approaching and detonating grenade. Our method provides continuous real-time feedback, and was retrospectively judged by our subjects as being intuitive.

## Methods

### Ethics statement

This work has been approved in advance by the Ethics Committee of the German Sport University. All subjects signed an informed consent statement before participating.

**Figure 1:**
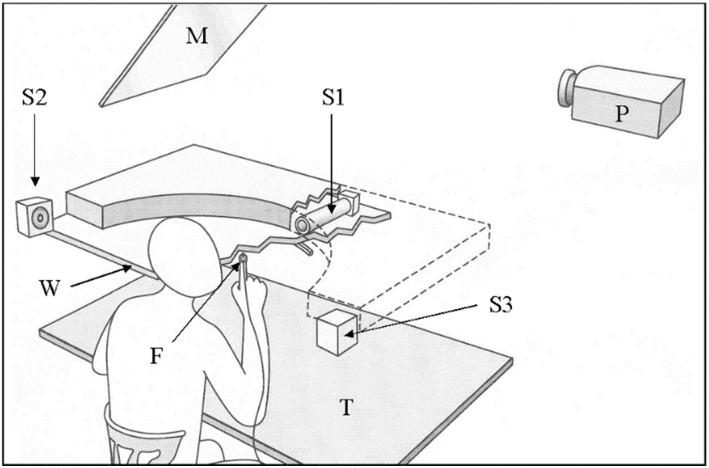
Scheme of the experimental apparatus. Subjects rested their chin above a horizontal panel (W). Auditory targets were six miniature sound sources (S1), arranged semi circularly at 12 deg intervals. Visual targets were projected (P) via a mirror (M) onto W, just in front of the sound sources. Two sound boxes to the left (S2) and right (S3) of the subject informed about directional errors. Movement of the index finger (F) was registered by an electromagnetic 3D tracking system. The other arm rested on a table (T) underneath W.

41 male and 31 female subjects aged 22.8±2.7 years participated. Subjects were right-handed, healthy, and had no prior experience with adaptation research. As shown in Figure 1, subjects sat at a table while visual (light dots of 1.5 cm radius) or auditory targets (mix of 0.45, 1.35, 2.30 and 3.20 kHz sound waves) were presented 36 cm ahead. Visual targets were projected onto the tabletop, and auditory targets emanated from miniature loudspeakers hidden from view by a horizontal panel and a vertical fabric screen. Targets of either modality were presented in balanced order at ±30, ±18, and ±6 deg about straight-ahead.

Subjects pointed to each target below the panel, using their index fingertip. Each response started from a wooden dowel underneath the subjects’ chin, proceeded radially until impact with a semicircular barrier underneath the target array, and then continued along that barrier until the finger reached the required position. The target was then extinguished, prompting subjects to return their finger to the dowel. The next target was presented 100 to 200 ms after that, etc. Index fingertip position was registered by an electromagnetic 3D tracking system with a resolution of 1 mm and 17 ms, and could be used to provide visual or auditory feedback about the ongoing response. Experiment A used either visual targets and visual feedback OR auditory targets and auditory feedback. Visual feedback was displayed as a cursor on the tabletop, and auditory feedback as a whistling sound from a loudspeaker to the subjects’ left when the finger deviated more than 2 deg counter-clockwise from the required direction, or from a loudspeaker to the subjects’ right when the finger deviated more than 2 deg clockwise; whistle frequency (1337 Hz) was modified by +300 Hz per 1 deg of deviation, and was replaced by the sound of an explosion once the finger reached the target area.

A different type of visual feedback was used in Experiment B: 3 rows of 11 arrows each were projected onto the table when the finger deviated from the required direction by more than 2 deg. The arrows were distributed across the whole table and their tips pointed to the right when the finger deviated to the left; they pointed to the left when the finger deviated to the right. The arrows were computer-generated and then displayed via a beamer, which allowed us to control the arrows’ color in dependence on the pointing error: the color changed from white to dark brown as finger direction approached target direction. This indirect feedback differs from the spatially coded visual feedback typically used in adaptation experiments; instead, it resembles the indirect auditory feedback presented in Experiment A.

An experiment lasted 45 minutes and was subdivided into episodes of 45 s duration (26 movements), separated by rest breaks of 5 s. Subjects were instructed to point quickly and accurately at each target and back, in a straight line. They practiced the task in one episode with and one episode without feedback, using the hand and sensory modality they would subsequently use for adaptation. Data acquisition then started with three baseline episodes where feedback was available, using again the same hand and modality; since performance was similar in all three episodes, they were treated as a single episode for data analysis. The baseline phase *with* feedback was followed by the baseline phase *without* feedback; in one episode each, subjects pointed with their right hand to visual, their right hand to auditory, their left hand to visual and their left hand to auditory targets. The order of these four conditions was counterbalanced across subjects. The subsequent adaptation phase consisted of 20 episodes with 30 deg rotated visual or auditory feedback. In Experiment A, subjects from group VR (n = 24) pointed with their right hand to visual targets under leftward-rotated visual feedback, group VL (n = 12) pointed with their left hand to visual targets under rightward-rotated visual feedback, group AR (n = 12) with their right hand to auditory targets under leftward-rotated auditory feedback, and group AL (n = 12) with their left hand to auditory targets under rightward-rotated auditory feedback. The larger sample size of VR is incidental: the data were collected for an earlier study with n = 24 (Bock and Schmitz, 2013) and were re-used for the present purposes; we hesitated to discard half of the data just to keep the group sizes equal. In Experiment B subjects (n = 12) pointed with their right hand to visual targets under leftward-rotated visual feedback. Experiment A and B concluded with the aftereffect phase which replicated the baseline phase without feedback, except for interleaved refresh episodes that mimicked the preceding adaption episodes. Like in the baseline phase, the order of conditions was counterbalanced in the aftereffect phase. Note that baseline and aftereffect phases included all four hand-modality combinations, but the adaptation phase included only a single one.

An interactive computer routine determined the directional error of each response as angular difference between response and target direction 166 ms after response onset, i.e., before feedback-based corrections could occur. We then calculated the mean directional errors of each episode and subject, and normalized them by subtracting the homologous (same hand and target modality) baseline values. The outcome was submitted to analyses of variance (ANOVAs), after data from group VL and AL were sign-reversed to facilitate comparisons of left- and rightward rotations. ANOVA for the baseline phase of Experiment A included the between-factor Modality (visual/auditory) and within-factor Feedback (with, without); ANOVA of the adaptation phase included the between-factors Adapted Hand (right, left) and Adapted Modality (visual, auditory) and the within-factor Episode; ANOVA on baseline-adjusted values of the aftereffect-phase used the between factors Adapted Hand and Adapted Modality and the within-factors Tested Hand (same as adapted, other) and Tested Modality (same as adapted, other); and a similar ANOVA comparing unadjusted values of the aftereffect phase with values from the baseline-phase further included the between-factor Phase (baseline-phase, aftereffect phase). Note that effects tested in the ANOVA of above-baseline aftereffects became interactions with Phase in the ANOVA comparing unadjusted aftereffects with baseline-values and the statistical results were exactly the same. Other ANOVAs compared Experiment B to groups VR and AR of Experiment A, using the between-factor Group and the same within-factors as above. Sphericity assumption was scrutinized with Mauchley’s test [14]; if significant, results were adjusted according to Greenhouse-Geisser [15]. Newman-Keuls post hoc test was chosen for post hoc comparisons.

The data of this study can be downloaded as Supporting Information file (Data S1) and obtained from the first author via email.

## Results

### Experiment A

**Figure 2:**
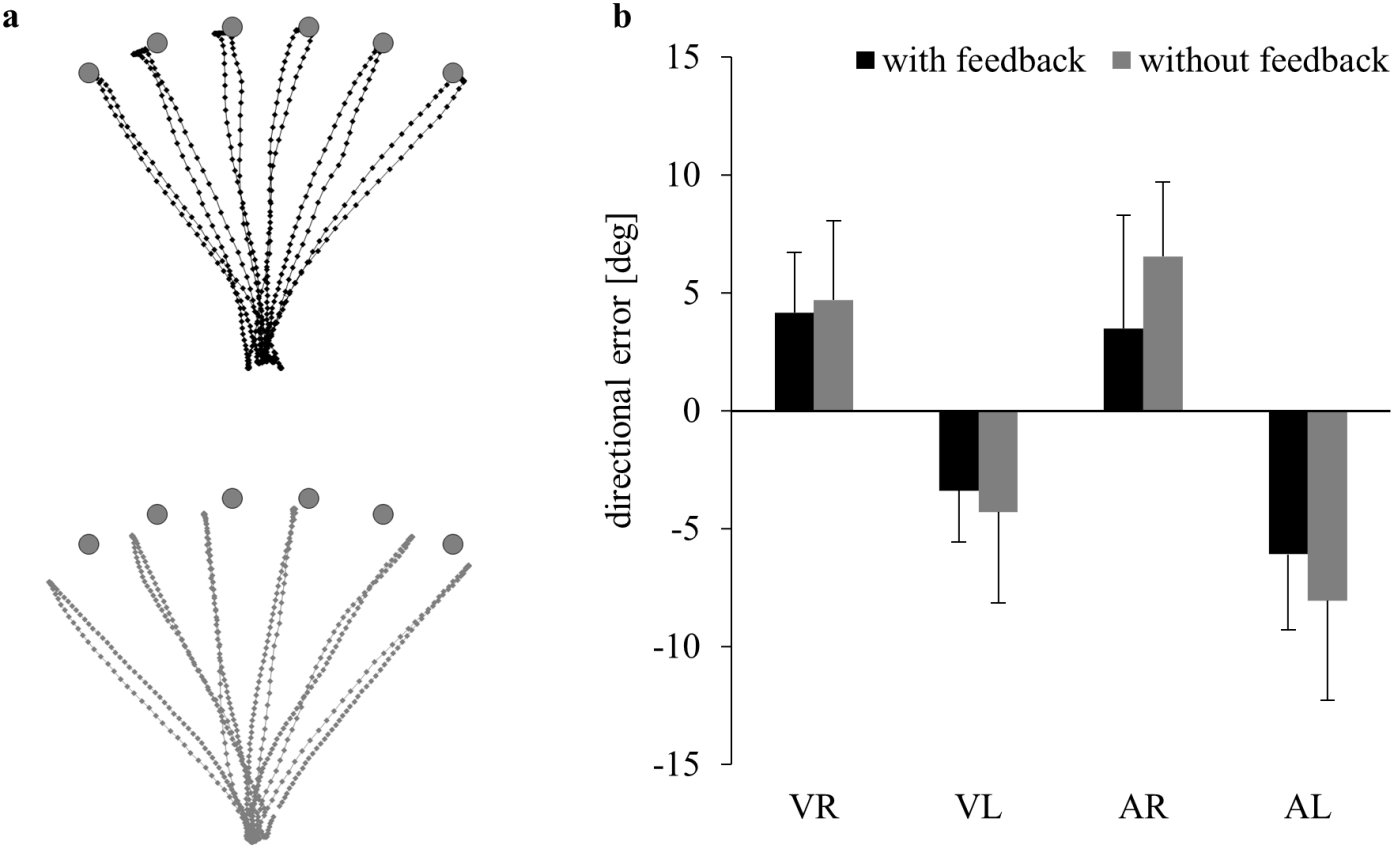
Movements from the baseline phase, executed with (black) or without feedback (grey). a) Original finger paths towards auditory targets with and without auditory feedback. b) Means and standard deviations of directional errors across subjects pointing with the to-be-adapted hand and the to-be-adapted modality (R = right, L = left, V = visual, A = auditory).

Sample registrations of pointing movements towards auditory targets from the baseline phase of one subject are shown in Figure 2a. Movements were accurate when auditory feedback was provided, and were somewhat less accurate when this feedback was removed. The aggregated data in Figure 2b reveal a similar pattern for all groups, i.e., movements with either hand to targets in either modality were somewhat more accurate with than without feedback. Accordingly, ANOVA yielded a significant effect of Feedback (F(1,56) = 13.50, p<0.001, ɳ^2^_p_ = 0.19). We also found significant effects of Modality (F(1,56) = 5.54, p<0.05, ɳ^2^_p_ = 0.09), as movements to visual targets were more accurate than those to auditory ones, and a significance of Feedback×Modality (F(1,56) = 4.15, p<0.05, ɳ^2^_p_ = 0.07), as the difference between modalities was smaller with than without feedback. Post-hoc decomposition revealed a significant modality difference only without feedback (p<0.01), not with feedback (p>0.05).

**Figure 3:**
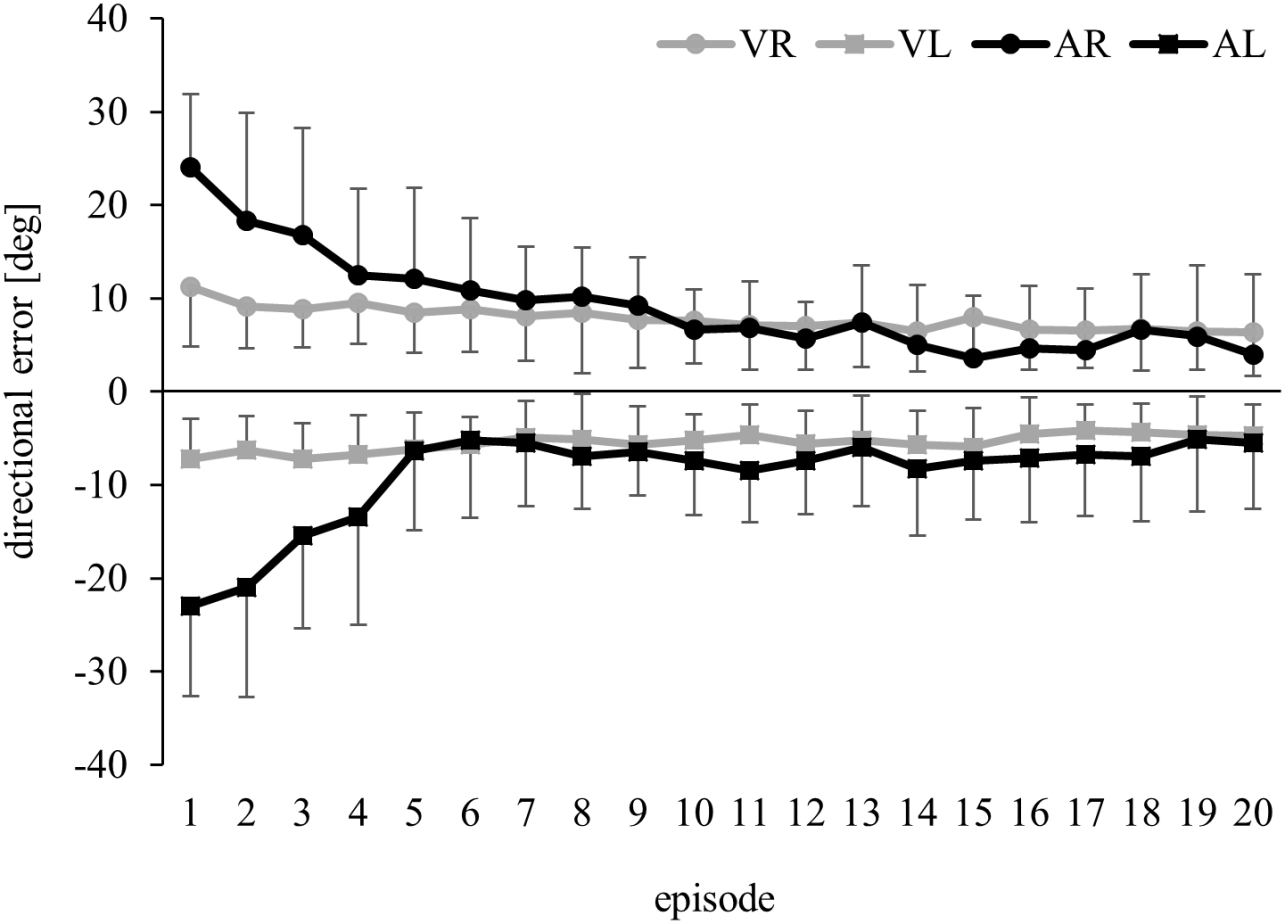
Means and standard deviations of directional errors in the adaptation phase, from subjects pointing with their right (R) or left hand (L) to visual (V) or auditory (A) targets. Subjects pointing with their right hand were exposed to a +30 deg rotation of feedback and their responses therefore deviated by up to +30 deg from the target direction. Subjects pointing with their left hand were exposed to a −30 deg rotation, and their responses therefore deviated by up to −30 deg from the target direction.

Figure 3 illustrates the above-baseline errors during the adaptation phase of all groups. ANOVA confirmed that errors gradually decreased (Episode: F(19,1064) = 29.47, p<0.001, ɳ^2^_p_ = 0.34), much faster so for visual than for auditory adaptation (Episode×Adapted Modality: F(19,1064) = 14.49, p<0.001, ɳ^2^_p_ = 0.21). Post-hoc tests revealed differences between modalities only for the first four adaptation episodes (p<0.05) and although visual adaptation progressed within a few movements, the first episode of visual adaptation still differed significantly from the last two episodes (p<0.05).

**Figure 4:**
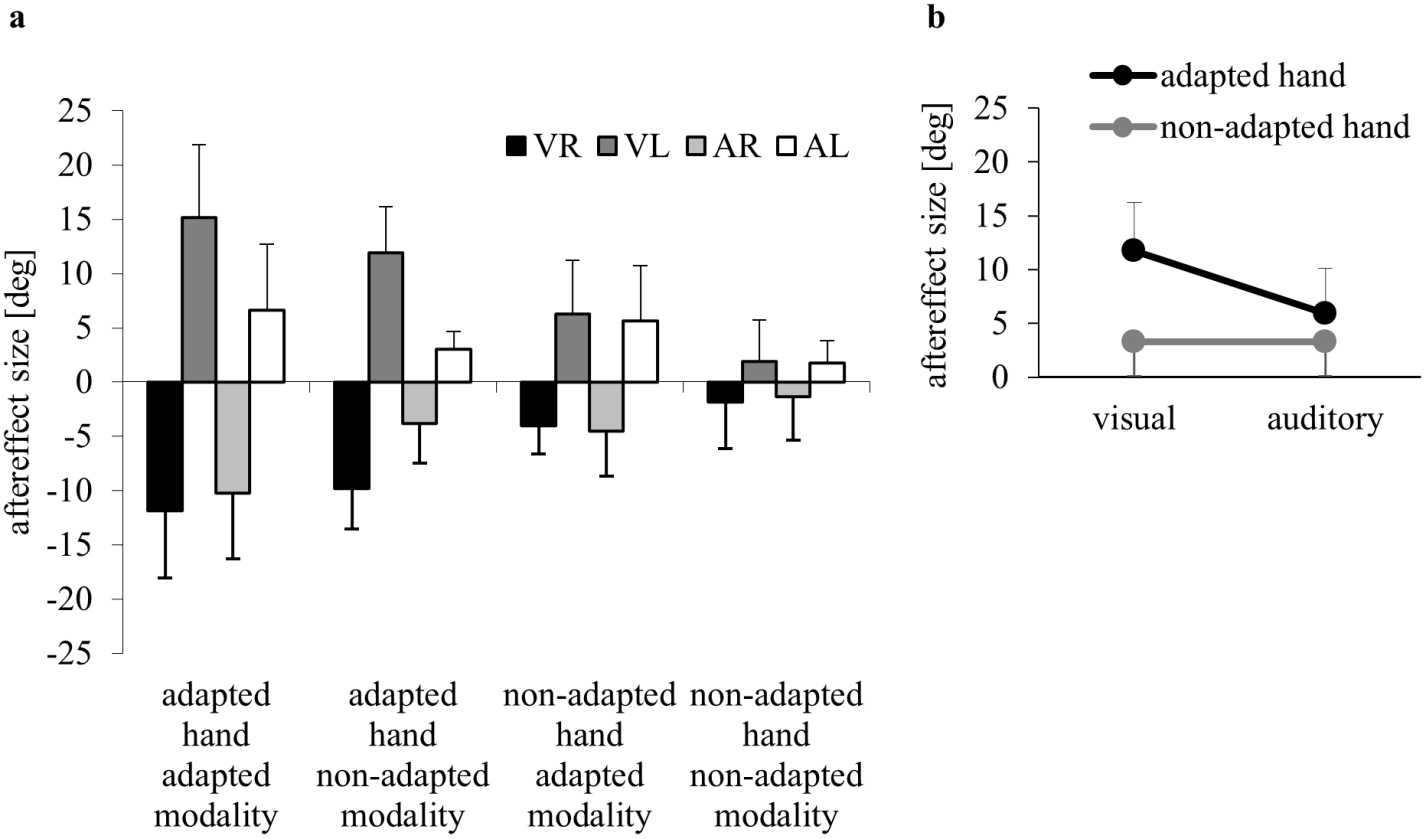
Aftereffects. a) Means and standard deviations of directional errors in aftereffect episodes, from subjects that had adapted the right (R) or left hand (L) to rotated visual (V) or auditory feedback (A). b) Means and standard deviations of the aggregated aftereffects for the adapted and the non-adapted hand in the visual and auditory groups. Note that values of groups VR and AR had been inverted.

Above-baseline aftereffects are depicted in Figure 4a. ANOVA confirmed that they were significantly larger in the previously adapted than in the non-adapted modality (Tested Modality: F(1,56) = 50.32, p<0.001, ɳ^2^_p_ = 0.47), and significantly larger with the previously adapted than with the non-adapted hand (Tested Hand: F(1,56) = 95.45, p<0.001, ɳ^2^_p_ = 0.63). Significance was also yielded for Adapted Modality (F(1,56) = 14.59, p<0.001, ɳ^2^_p_ = 0.21) and Adapted Modality×Tested Hand (F(1,56) = 27.19, p<0.001, ɳ^2^_p_ = 0.33). The latter two findings are illustrated in Figure 4b: the advantage of the adapted over the non-adapted hand was much larger following visual (VR, VL) than following auditory adaptation (AR, AL).

ANOVA on unadjusted aftereffects confirmed that changes from baseline- to aftereffect-phase were significant (Phase: F(1,56) = 218.08, p<0.001, ɳ^2^_p_ = 0.80). Post-hoc decompositions of interactions with Phase yielded significance for aftereffects of the adapted and the non-adapted modality, and of the adapted as well as the non-adapted hand after visual and after auditory adaptation (all p<0.001). Thus, even the aftereffects of the non-adapted systems were significant.

### Experiment B

**Figure 5:**
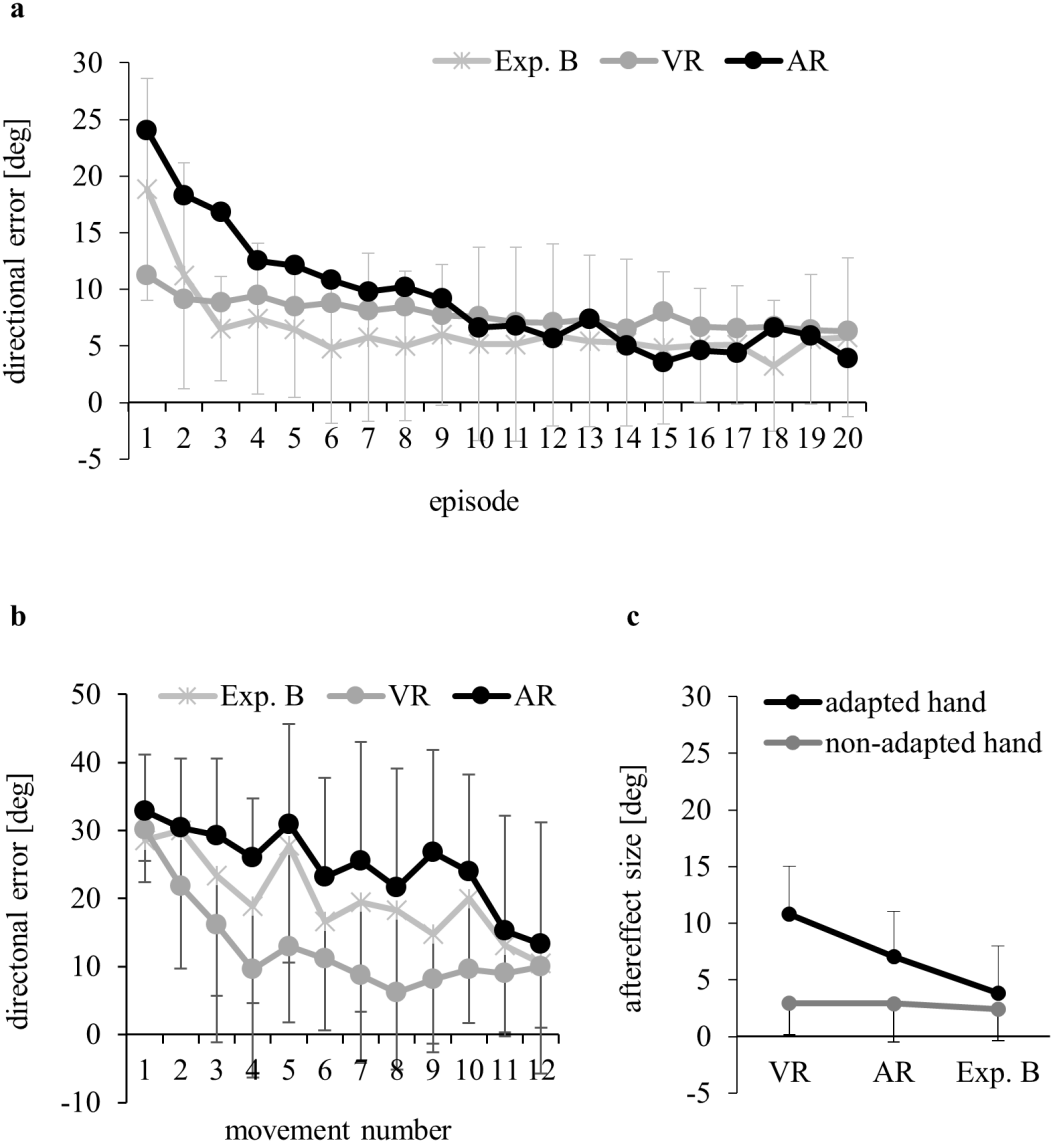
Means and standard deviations of directional errors, from subjects adapting to rotated indirect visual feedback (Exp. B), direct visual feedback (VR from Exp. A, without standard deviations in a)) or indirect auditory feedback (AR from Exp. A, without standard deviations in a)). Data come from a) all episodes of the adaptation phase, b) the first 12 movements of the adaptation phase and c) the aftereffect phase.

Figure 5a illustrates the time-course of adaptation with indirect visual feedback. Two-way ANOVA of above-baseline errors yielded no significance for Group (F(2,45) = 1.03, p>0.05, ɳ^2^_p_ = 0.05), but significance for Episode (F(19,855) = 25.27, p<0.001, ɳ^2^_p_ = 0.30) and Episode×Group (38,855) = 5.26, p<0.001, ɳ^2^_p_ = 0.19). Post-hoc decomposition revealed that during the first adaptation episode, errors were smaller in group VR than in Exp. B (p<0.05), and during the first three adaptation episodes smaller in Exp. B than in group AR (all p<0.05). Figure 5b shows that these group differences emerged already during the initial few movements of the first adaptation episode.

Fig. 5c compares the above-baseline aftereffects of Exp. B with those from groups VR and AR. ANOVA confirmed significantly larger aftereffects in the previously adapted than in the non-adapted modality (Tested Modality: F(1,45) = 23.37, p<0.001, ɳ^2^_p_ = 0.34), and significantly larger aftereffects with the previously adapted than with the non-adapted hand (Tested Hand: F(1,45) = 40.05, p<0.001, ɳ^2^_p_ = 0.47). We also found significance for Group (F(2,45) = 7.64, p = 0.001, ɳ^2^_p_ = 0.25) and Group×Tested Hand (F(2,45) = 8.26, p<0.001, ɳ^2^_p_ = 0.27). As illustrated in Fig. 5c, the advantage of the adapted over the non-adapted hand was largest in VR, smaller in AR and smaller still in Exp. B.

ANOVA of unadjusted aftereffects confirmed the significance of differences between baseline- and aftereffect phase (Phase: F(1,45) = 142.37, p<0.001, ɳ^2^_p_ = 0.76). Post-hoc decompositions of interactions with Phase yielded significance for aftereffects of the adapted and non-adapted modality (p<0.001), as well as the adapted hand (p<0.001) and the non-adapted hand (p<0.05) in all groups.

## Discussion

The present work compares the adaptation of pointing movements in the visual and in the auditory modality. Data from the baseline phase confirm that our auditory paradigm was effective: subjects pointed with auditory feedback to auditory targets as accurately as they did with visual feedback to visual targets. Only when feedback was absent did auditory accuracy drop below visual accuracy. The latter finding seems at odds with an earlier study [11], which found no difference in accuracy when pointing to auditory targets with and without auditory feedback. In that study, however, accuracy didn’t change even when auditory feedback was laterally displaced, which suggests that the method for feedback delivery was ineffective in that earlier study.

Our data from the adaptation phase indicate that all subject groups eventually compensated for the imposed lateral distortion; the initial speed of improvement was highest with direct visual feedback, lower with indirect visual feedback, and lowest with indirect auditory feedback, but all groups reached similar asymptotes later during the adaptation phase. Different adaptation speeds might be explained by several factors as for example number and spacing of targets [16]–[17], but also by cognitive strategies [18]. The differences observed in the present study can’t be attributed to a different number, location or timing of targets, nor to different verbal instructions, since those were comparable in all subject groups. Rather, the differences of adaptation speed seem to be directly related to the type of feedback. We interpret this pattern of findings as evidence that indirect auditory feedback had a higher computational demand than indirect visual feedback which, in turn, had a higher computational demand than direct visual feedback. If so, direct visual feedback would leave most computational resources available for adaptive improvement, indirect visual feedback would leave less, and indirect auditory feedback would leave least resources available for adaptation.

It is generally accepted that subjects’ performance during the adaptation phase is governed by two processes, adaptive recalibration and workaround strategies, while performance during the aftereffect phase reflects adaptive recalibration alone [1], [19]. An analysis of aftereffects therefore provides more direct insights into the principles of adaptive recalibration than an analysis of the adaptation phase. The purpose of the present study was to scrutinize two possible interpretations of our previous findings regarding aftereffects [7]: are aftereffects larger when they are tested with visual rather than with auditory targets, or larger when they are tested with targets from the previously trained rather than an untrained modality (see Introduction)?

We found aftereffects to be significant. For the non-adapted hand, they were only 37% of the size of the aftereffects for the previously adapted hand, in accordance with the well-known fact that intermanual transfer is incomplete. We further found aftereffects to be significant in the previously adapted as well as the non-adapted modality. This finding is in accordance with results from studies reporting visually induced changes in sound localization [20] or audiomotor aftereffects of adaptation to prisms [21] or rotated visual feedback [4]. But in contrast to the latter study we found transfer between sensory modalities to be incomplete: Aftereffects for the non-adapted modality were only 59% of those for the previously adapted modality. This discrepancy might be explained by the order of aftereffect tests: Kagerer and Contreras-Vidal [4] tested the non-adapted modality first. Thus, aftereffects of the adapted modality might have been underestimated due to de-adaptation. In our study, the order of aftereffect tests was balanced across subjects and interleaved with refresh episodes to minimize de-adaptation. Therefore we conclude that transfer between sensory modalities is incomplete as well.

Aftereffects following auditory adaptation were 65% of those following visual adaptation in Exp. A, at least when the previously adapted hand was tested. The latter finding seems to indicate that visual adaptation is more effective than auditory adaptation; however, Exp. B places the data into a different perspective: aftereffects with the previously adapted hand were largest following adaptation with direct visual feedback, smaller for indirect acoustic feedback, and smaller still for indirect visual feedback. This suggests that the method of feedback delivery - direct versus indirect - is more critical than the modality of that feedback.

Coming back to the two alternative interpretations of our previous study [7], we found no evidence for a supremacy of visual over auditory adaptation, but we did find evidence for an incomplete transfer of adaptation between sensory modalities. In addition, we found that adaptation is more efficient with direct rather than indirect feedback, and therefore posit that auditory adaptation would be just as efficient as visual adaptation if auditory feedback could be delivered in the same direct fashion as visual feedback normally is.

The results of the present study might amend parts of a recently published, conceptual model on sensorimotor adaptation [6], which states that adaptive mechanisms receive un-weighted sensory input. The results on intermodal transfer suggest that sensory input is weighted, and the weight depends on whether a modality had previously adapted or not. If weights are subject to change, the amended model might also explain a further result from Kagerer and Contreras-Vidal [4] who reported that visual but not auditory aftereffects persisted from aftereffect- to retention-test. Incomplete intermanual transfer confirms the prediction of lower output weights for non-adapted compared to adapted effectors. Our findings from Exp. B might further imply that feedback type influences the output weight of the adapted effector as well.

## Supporting Information

Data S1 **Original data.**
(XLSX)
